# Attenuation of Nonsense-Mediated mRNA Decay Enhances In Vivo Nonsense Suppression

**DOI:** 10.1371/journal.pone.0060478

**Published:** 2013-04-10

**Authors:** Kim M. Keeling, Dan Wang, Yanying Dai, Srinivasan Murugesan, Balachandra Chenna, Jeremy Clark, Valery Belakhov, Jeyakumar Kandasamy, Sadanandan E. Velu, Timor Baasov, David M. Bedwell

**Affiliations:** 1 Department of Microbiology, University of Alabama at Birmingham, Birmingham, Alabama, United States of America; 2 Department of Genetics, University of Alabama at Birmingham, Birmingham, Alabama, United States of America; 4 Department of Chemistry, University of Alabama Birmingham, Birmingham, Alabama, United States of America; 5 The Edith and Joseph Fischer Enzyme Inhibitors Laboratory, Schulich Faculty of Chemistry, Technion-Israel Institute of Technology, Haifa, Israel; University of North Carolina, United States of America

## Abstract

Nonsense suppression therapy is an approach to treat genetic diseases caused by nonsense mutations. This therapeutic strategy pharmacologically suppresses translation termination at Premature Termination Codons (PTCs) in order to restore expression of functional protein. However, the process of Nonsense-Mediated mRNA Decay (NMD), which reduces the abundance of mRNAs containing PTCs, frequently limits this approach. Here, we used a mouse model of the lysosomal storage disease mucopolysaccharidosis I-Hurler (MPS I-H) that carries a PTC in the *Idua* locus to test whether NMD attenuation can enhance PTC suppression *in vivo*. *Idua* encodes alpha-L-iduronidase, an enzyme required for degradation of the glycosaminoglycans (GAGs) heparan sulfate and dermatan sulfate. We found that the NMD attenuator NMDI-1 increased the abundance of the PTC-containing *Idua* transcript. Furthermore, co-administration of NMDI-1 with the PTC suppression drug gentamicin enhanced alpha-L-iduronidase activity compared to gentamicin alone, leading to a greater reduction of GAG storage in mouse tissues, including the brain. These results demonstrate that NMD attenuation significantly enhances suppression therapy *in vivo*.

## Introduction

Nonsense suppression drugs reduce the efficiency of translation termination at in-frame premature termination codons (PTCs; nonsense mutations), thereby allowing ribosomes to resume translation elongation and generate a full-length protein [1], [2]. While PTC “readthrough” has been shown to restore functional protein in a range of disease models [1]–[4], recent clinical trial results suggest that current suppression therapy approaches may not restore enough protein function to provide a clear therapeutic benefit for some diseases [5]–[12]. Suppression therapy is frequently limited by nonsense-mediated mRNA decay (NMD), a conserved eukaryotic cellular pathway that targets PTC-containing mRNAs for degradation [13]–[15]. We hypothesize that attenuating NMD to increase the abundance of PTC-containing mRNAs will restore higher levels of functional protein produced by PTC suppression, thus providing a greater therapeutic benefit.

UPF1 is a phosphoprotein that is essential for NMD function. The UPF1 phosphorylation cycle represents a potential pharmacological target for NMD attenuation [16], [17]. The kinase SMG1, UPF1, and the release factor complex that mediates translation termination form the SURF complex at PTC-bound ribosomes to initiate NMD [18]–[20]. If a PTC-bound SURF complex interacts with a downstream exon junction complex, SMG1 phosphorylates UPF1 at multiple residues, which marks the transcript for decay. Subsequently, the SMG5/7 complex recruits the PP2A phosphatase to dephosphorylate and recycle UPF1, while the mRNA decay machinery subsequently degrades the PTC-containing transcript [21], [22]. Several drugs have been found to attenuate NMD by inhibiting the UPF1 phosphorylation cycle. SMG1 kinase inhibitors, including caffeine, wortmannin, and LYS294002, prevent UPF1 phosphorylation [18], [23]. In contrast, NMDI-1 blocks UPF1 dephosphorylation by disrupting the interaction between SMG5 and phospho-UPF1 [24].

In this study, we examined whether NMD attenuation with compounds that disrupt the UPF1 phosphorylation cycle enhanced PTC suppression in the *Idua* ^W392X^ knock-in mouse. This mouse carries a genomic nonsense mutation in the *Idua* locus that induces NMD of the *Idua* mRNA, abrogates α-L-iduronidase function, and serves as a model for the lysosomal storage disease mucopolysaccharidosis type I-Hurler (MPS I-H) [25], [26]. MPS I-H is an ideal disease model to investigate suppression therapy and NMD attenuation for several reasons. First, nonsense mutations are present in ∼75% of MPS I-H patients [27]. Second, NMD has been reported to reduce *IDUA* mRNA levels in MPS I-H patients that carry nonsense mutations [28]. Third, MPS I-H has a low threshold for correction, since <1% of wild-type iduronidase function can significantly moderate the clinical phenotype [29], [30]. In the current study, we found that UPF1 phosphorylation cycle inhibitors increased steady-state *Idua* ^W392X^ RNA levels *in vitro* and *in vivo*. In agreement with our hypothesis, we also found that co-administration of the NMD attenuator NMDI-1 with a subset of PTC suppression drugs alleviated MPS I-H biochemical defects to a greater extent than suppression therapy alone both *in vitro* and *in vivo*. These results provide the first *in vivo* evidence that PTC suppression efficacy can be improved by attenuating NMD efficiency.

## Results

### NMDI-1 is an efficient NMD attenuator

**Figure 1:**
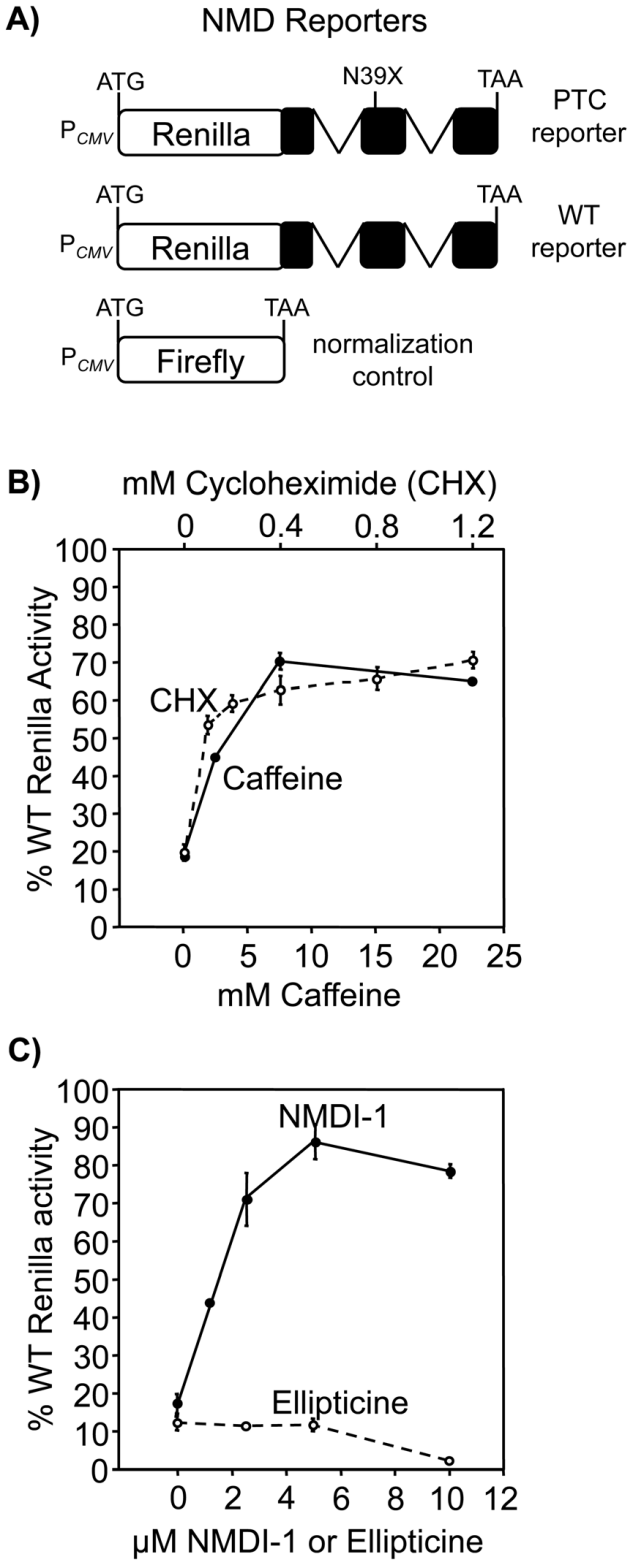
Caffeine and NMDI-1 attenuate NMD. A) HeLa cells expressing luciferase-based NMD reporters [32] were used to monitor the effect of various drugs on NMD efficiency, which was expressed as the normalized N39X *Renilla* expression relative to WT ×100 (% WT *Renilla* activity). B) The effect of cycloheximide (CHX) (open circles, dashed line) and caffeine (closed circles, solid line) on NMD. C) The effect of ellipticine (open circles, dashed line) and NMDI-1 (closed circles, solid line) on NMD. The data shown are expressed as the mean +/− sd of a representative assay performed in triplicate.

Caffeine and NMDI-1 attenuate NMD by blocking the UPF1 phosphorylation cycle (**Figure S1A**). Caffeine inhibits the SMG1 kinase that phosphorylates UPF1 [23], [31], while NMDI-1 blocks the interaction between UPF1 and SMG5, which prevents the recruitment of the PP2A phosphatase to dephosphorylate UPF1 [24]. To compare the relative efficacy of these two drugs to attenuate NMD, we initially used luciferase NMD reporters expressed in HeLa cells (**Figure 1A**). The reporters consist of *Renilla* luciferase fused to WT β-globin or β-globin containing a PTC (N39X) that induces NMD [32]. The amount of *Renilla* activity expressed from the N39X reporter relative to the WT control correlates with mRNA abundance and NMD efficiency. In untreated cells, N39X *Renilla* activity was ∼15% of the WT control (**Figure 1B, C**). Treatment with caffeine or the translation inhibitor cycloheximide elevated N39X *Renilla* activity to ∼70% of the WT control, indicating that both compounds effectively inhibited NMD (**Figure 1B**). NMDI-1 treatment increased N39X *Renilla* activity to ∼85% of the WT control (**Figure 1C**). In contrast, ellipticine, an antineoplastic compound [33] that is structurally related to NMDI-1 **(Figure S1B)**, did not inhibit NMD. Additional analyses also showed that NMDI-1 does not inhibit protein synthesis or directly suppress PTCs in a reporter construct that is not subject to NMD (**Figure S2A, B**). These results confirm that both caffeine and NMDI-1 attenuate NMD in mammalian cells, and establish that NMDI-1 is effective at much lower concentrations.

### NMDI-1 attenuates NMD in MEFs

**Figure 2:**
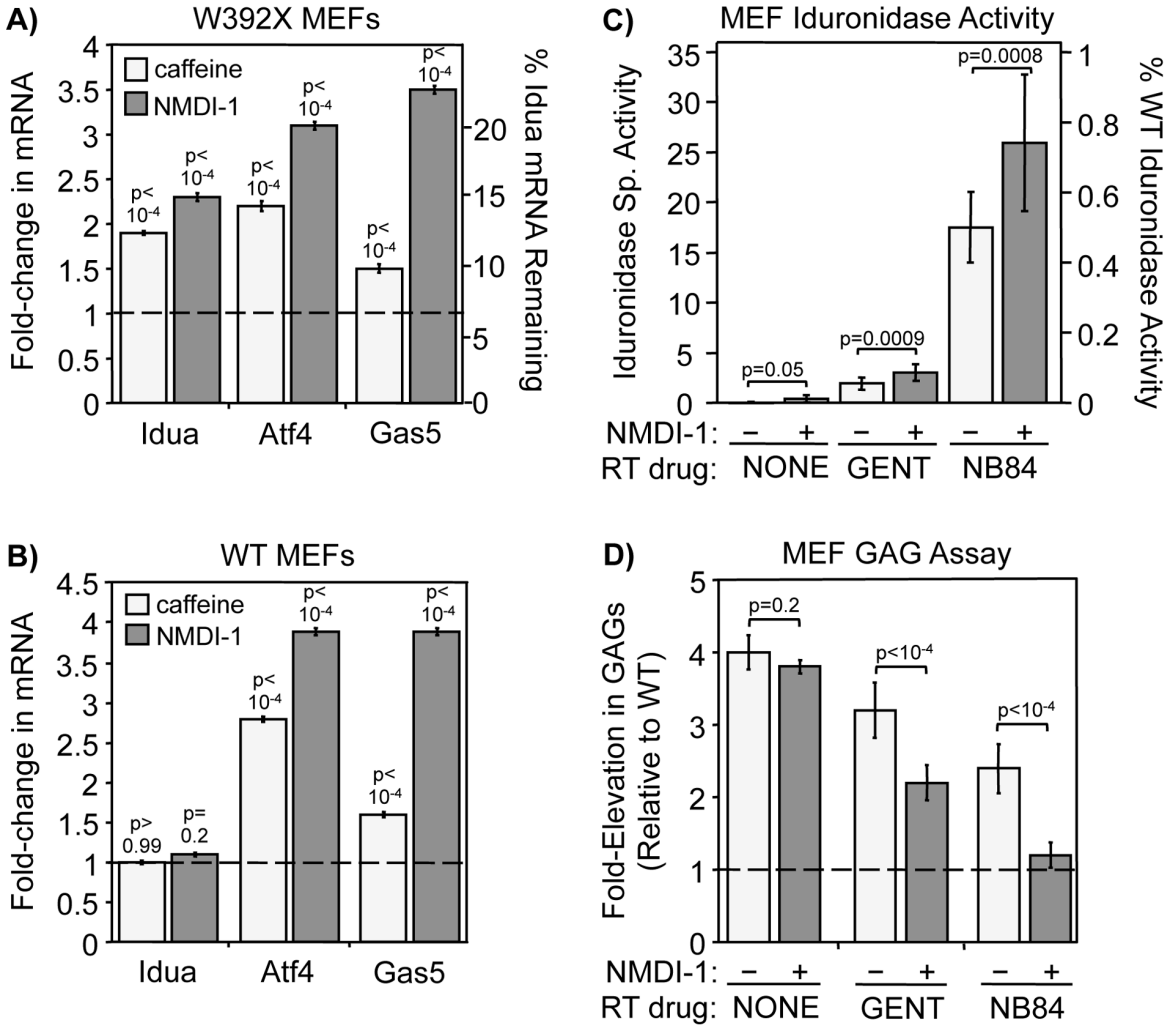
NMD attenuators reduce NMD efficiency and enhance PTC suppression in *Idua* ^W392X^ MEFs. MEFs were cultured +/− 7.5 mM caffeine for 4 hr or 5 μM NMDI-1 for 24 hr prior to RNA isolation. RT-qPCR was performed to quantitate NMD substrate steady state levels. A) Quantitation of *Idua, Atf4*, and *Gas5* mRNAs in treated relative to untreated *Idua* ^W392X^ MEFs (indicated by the dashed line  = 1). B) Quantitation of *Idua*, *Atf4*, and *Gas5* mRNAs in treated relative to untreated WT MEFs (indicated by dashed line = 1). For A & B, results were normalized to 5S rRNA. Similar results were obtained when NMD substrates were normalized to 18S rRNA or *Rpl13a* (Figure S3). All data are the mean +/− sd of at least 3 independent experiments performed with ≥6 replicates (n = 3). *p* values above the columns compare treated to untreated MEFs. C) α-L-iduronidase activity in *Idua* ^W392X^ MEFs cultured without readthrough (RT) drugs, with gentamicin (GENT), or with NB84 in the absence (−) or presence (+) of NMDI-1. The data shown are the mean +/− sd of 3 independent experiments performed in triplicate (n = 3). D) Sulfated GAGs were quantitated in *Idua* ^W392X^ MEFs cultured without readthrough drugs, with gentamicin (GENT), or with NB84 in the absence (−) or presence (+) of NMDI-1. The dashed line represents the WT GAG level. The data shown are expressed as the mean +/− sd of 3 independent experiments performed at least in quadruplicate (n = 3). For C & D, *p* values above the brackets compare NMDI-1 treated cells versus untreated controls.

We next asked whether NMDI-1 and caffeine inhibit decay of endogenous NMD substrates in primary mouse embryonic fibroblasts (MEFs) derived from homozygous WT and *Idua* ^W392X^ mice. The abundance of three transcripts subject to NMD (*Idua, Aft4, Gas5*) was quantitated in MEFs treated with NMD attenuators (relative to untreated controls). *Idua* encodes α-L-iduronidase and the *Idua* ^W392X^ MEFs carry an in-frame PTC within the *Idua* open reading frame (ORF) that elicits NMD [25]. In untreated *Idua* ^W392X^ MEFs, NMD reduced *Idua* mRNA levels to 6% of WT MEF levels (**Figure 2A**). *Idua* mRNA levels in *Idua* ^W392X^ MEFs increased 1.9-fold following caffeine treatment and 2.3-fold after NMDI-1 treatment, corresponding to 11% and 15% of WT *Idua* levels, respectively. *Idua* is not an NMD substrate in WT MEFs, and its level remained unchanged when these cells were treated with either caffeine or NMDI-1 (**Figure 2B**). While mRNAs containing nonsense mutations are often subject to NMD, a number of endogenous transcripts in their natural form are also NMD substrates. These include transcripts with upstream ORFs such as *Aft4*, or with long or intron-containing 3′ untranslated regions such as *Gas5* [34]–[36]. Therefore *Atf4* and *Gas5* mRNAs are subject to NMD in both WT and *Idua* ^W392X^ MEFs. In MEFs from both lines, *Aft4* mRNA was elevated 2.5-fold and 3.5-fold following caffeine and NMDI-1 treatment, respectively; while caffeine and NMDI-1 treatment increased *Gas5* mRNA levels 1.5-fold and 3.7-fold, respectively (**Figure 2A, B**). Together, these results indicate that both of these compounds partially inhibit NMD and increase the abundance of various NMD substrates.

### Attenuating NMD efficiency enhances PTC suppression in MEFs

We next determined whether NMD attenuation enhanced suppression of the W392X PTC in *Idua* ^W392X^ MEFs. To do this, we compared MPS I-H biochemical endpoints in cells treated with each suppression drug plus NMDI-1 to cells treated with the corresponding readthrough drug alone. We first monitored α-L-iduronidase specific activity. α-L-iduronidase activity was undetectable in untreated *Idua* ^W392X^ MEFs, but a trace level of α-L-iduronidase activity was detected after NMDI-1 treatment (**Figure 2C**). Gentamicin treatment increased α-L-iduronidase activity 4.5-fold more than NMDI-1. NB84, a synthetic aminoglycoside designed to have enhanced readthrough efficiency with less toxicity than conventional aminoglycosides such as gentamicin [37], restored 9-fold more iduronidase activity than gentamicin [25]. Notably, treatment of cells with either gentamicin or NB84 in conjunction with NMDI-1 enhanced α-L-iduronidase activity by an additional 50%, suggesting that NMD attenuation enhances PTC suppression.

Since abnormal glycosaminoglycan (GAG) accumulation is the primary biochemical defect associated with α-L-iduronidase deficiency, we next assessed whether the amount of α-L-iduronidase activity restored by PTC suppression was sufficient to decrease excess GAG storage in *Idua* ^W392X^ MEFs (**Figure 2D**). Consistent with the MPS I-H phenotype, GAGs in untreated *Idua* ^W392X^ MEFs were elevated 4-fold compared to WT MEFs [25]. NMDI-1 treatment alone did not reduce GAG accumulation. However, MEFs treated with gentamicin or NB84 alone showed 26% and 53% reductions in excess GAGs, respectively, compared to untreated controls. More robust 54% and 93% reductions in GAG storage were observed when NMD-1 was co-administered with gentamicin or NB84, respectively. These results indicate that NMDI-1 significantly enhanced the level of PTC suppression mediated by gentamicin and NB84.

### NMDI-1 attenuates NMD in Idua^W392X^ mice

**Figure 3:**
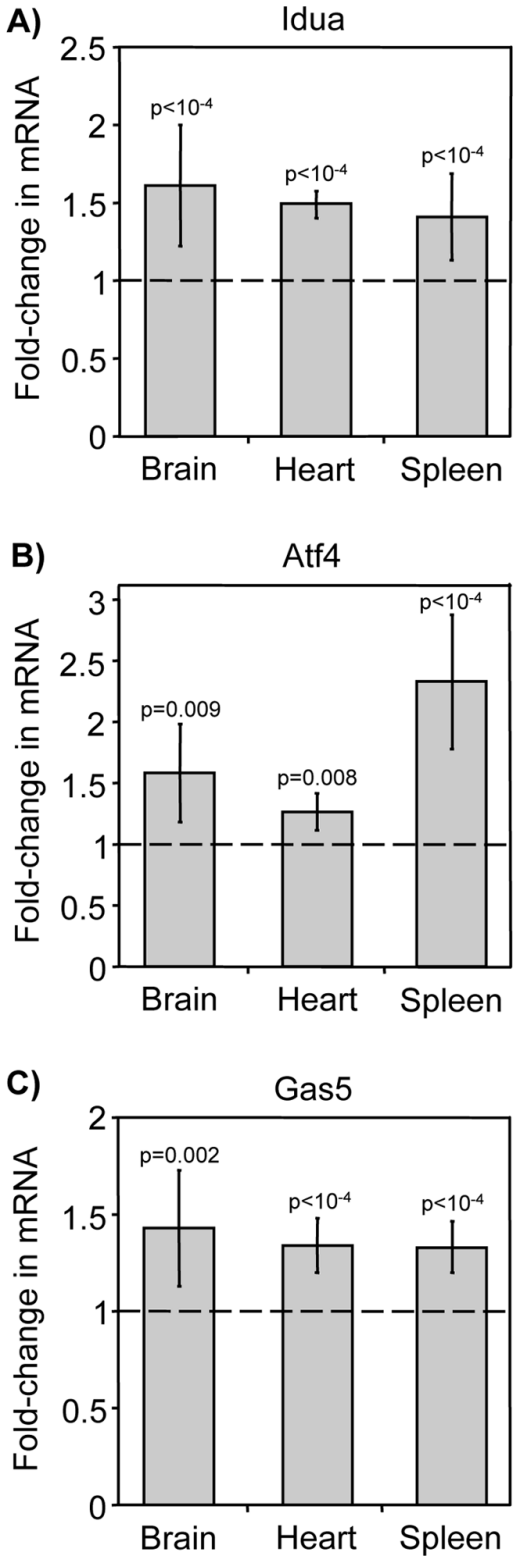
NMDI-1 increases the abundance of endogenous NMD substrates in *Idua* ^W392X^ mice. Homozygous *Idua* ^W392X^ mice were administered 5 mg/kg NMDI-1 for 3 days via once daily subcutaneous injections. After treatment, RNA was isolated from the brain, heart, and spleen and analyzed by RT-qPCR to determine NMD substrate steady state levels in NMDI-1 treated mice relative to untreated controls. The data shown are quantitation of the A) *Idua*, B) *Atf4*, and C) *Gas5* mRNAs normalized to 5S rRNA. Similar results were obtained when NMD substrates were normalized to 18S rRNA or *Rpl13a* (Figure S4). The data are expressed as the fold-change in RNA levels in *Idua* ^W392X^ mice treated with NMDI-1 relative to untreated *Idua* ^W392X^ mice (indicated by dashed line  = 1). All data are the mean +/− sd of values obtained from 3 mice per group, performed with ≥6 replicates (n = 3). *p* values above the columns compare treated to untreated mice.

We next evaluated the ability of NMDI-1 to attenuate NMD *in vivo*. We treated homozygous *Idua* ^W392X^ mice with NMDI-1 once daily for 3 days and quantitated the steady-state levels of the *Idua*, *Atf4*, and *Gas5* mRNAs in the brain, heart, and spleen (**Figure 3**). We found that the abundance of all three NMD substrates was significantly increased (relative to untreated controls). Among the three organs, *Idua* mRNA levels increased ∼1.5-fold, *Aft4* levels rose 1.3 to 2.3-fold, and *Gas5* levels increased ∼1.4-fold. No adverse side effects were observed in mice during short-term NMDI-1 administration (3 days). Mouse behavior was unaltered, no weight loss was observed, and urine creatinine levels remained unchanged. Histological analysis of mice treated with NMDI-1 also revealed no gross tissue abnormalities.

### NMDI-1 administration enhances PTC suppression in Idua^W392X^ mice

**Figure 4:**
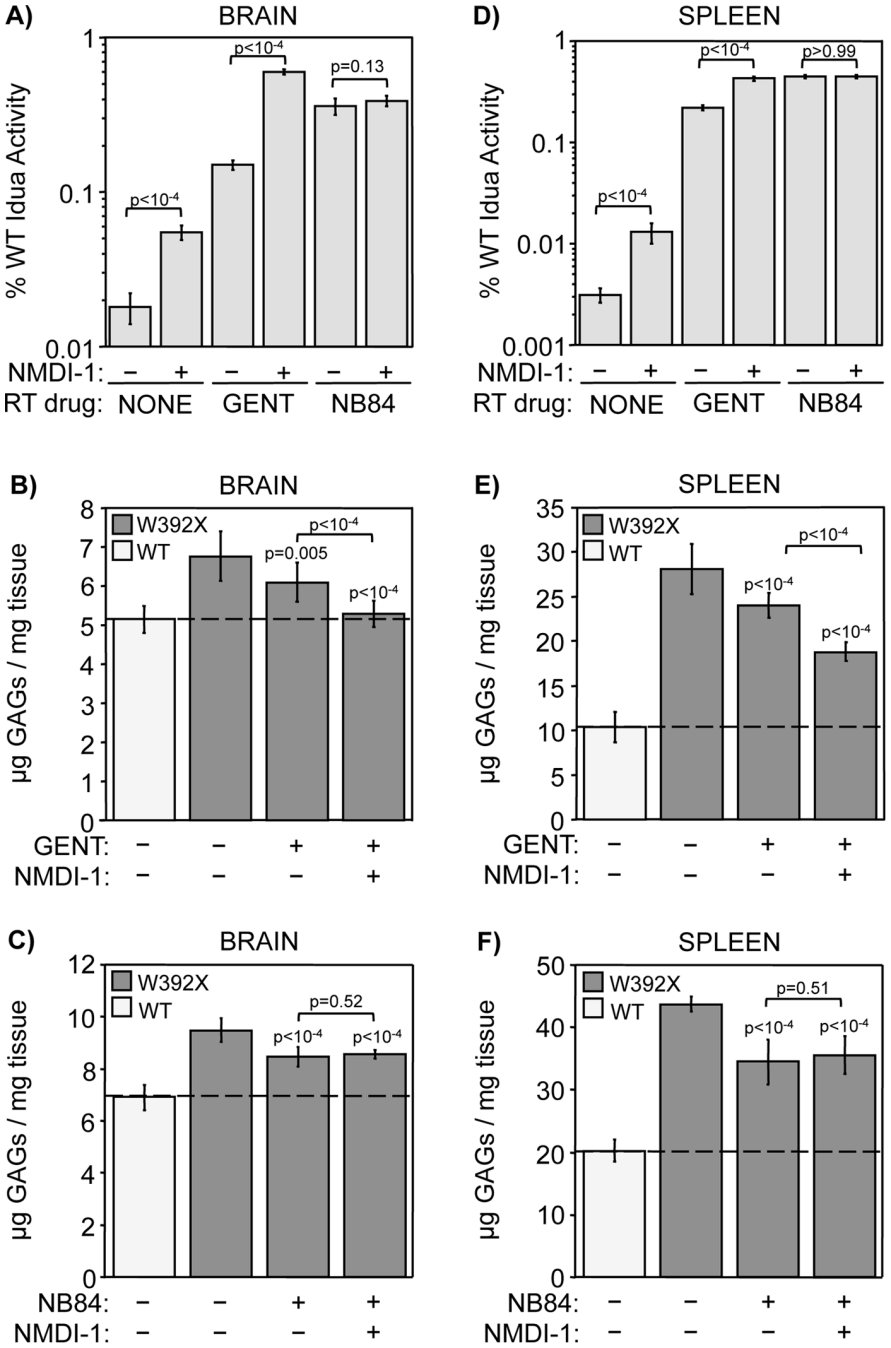
NMDI-1 co-administration with gentamicin, but not NB84, enhances PTC suppression in *Idua* ^W392X^ mice. Homozygous WT and *Idua* ^W392X^ mice were administered readthrough (RT) drugs gentamicin (GENT) or NB84 for 14 days without (−) or with (+) NMDI-1 administration during the final 3 days of treatment. α-L-iduronidase (Idua) specific activity was determined in A) brain and D) spleen. The data are expressed as the specific activity in the mutant mouse tissues relative to WT controls × 100 (% WT Idua Activity). Data in A & D are the mean +/− sd of values obtained from 3–4 mice per group, performed using ≥8 replicates (n = 3 or 4). *p* values above the brackets compare mice treated with NMDI-1 to those without NMDI-1 treatment. B, C, E, F) Sulfated GAG levels were quantitated in brain and spleen from WT mice (white bars) and from untreated and treated *Idua* ^W392X^ mice (gray bars). GAG levels were quantitated as micrograms GAGs per mg protein in B) brain and E) spleen from the gentamicin treatment group, or in C) brain and F) spleen from the NB84 treatment group. The dashed line represents the WT GAG level as a reference. Data in B, C, E, F are expressed as mean +/− sd of 15–18 assays from 5–6 mice for each experimental group (n =  5 or 6). *p* values above the columns compare treated versus untreated W392X mice. *p* values above the brackets compare mice co-treated with both RT drug and NMDI-1 compared to mice treated with RT drug alone.

To determine whether NMDI-1 enhanced PTC suppression *in vivo*, α-L-iduronidase activity was evaluated in the brain and spleen of *Idua* ^W392X^ mice treated with gentamicin or NB84 +/− NMDI-1 (**Figure 4A, D**). Gentamicin and NB84 were administered at a 30 mg/kg dose to 10-week old mice via once daily subcutaneous injections for 14 days. NMDI-1 was co-administered at a dose of 5 mg/kg via subcutaneous injections during the final three days of the 14-day treatment. Previous studies have indicated that as little as 0.1–1% of normal α-L-iduronidase activity can alleviate clinical symptoms of MPS I-H [29], [30]. In untreated *Idua* ^W392X^ mice, α-L-iduronidase activity in the brain and spleen was <0.02% of WT activity. NMDI-1 treatment alone led to a significant increase in α-L-iduronidase activity in the brain and spleen compared to untreated mice, but activity remained <0.1% of normal. Gentamicin administration alone restored 0.15% and 0.22% of WT α-L-iduronidase in the brain and spleen, respectively. NMDI-1 co-administration with gentamicin increased α-L-iduronidase activity 4-fold in the brain and 2-fold in the spleen compared to gentamicin treatment alone, resulting in α-L-iduronidase levels that were 0.60% and 0.42% of WT, respectively. NB84 treatment alone restored 0.36% and 0.45% of WT α-L-iduronidase activity in the brain and spleen, respectively. Surprisingly, no additional enhancement in activity was found when NMDI-1 was co-administered with NB84.

We next evaluated whether the level of α-L-iduronidase restored in *Idua* ^W392X^ mice by gentamicin or NB84 +/− NMDI-1 reduced excess GAG storage. We found that NMDI-1 treatment alone did not significantly reduce tissue GAG storage (**Figure S5**). However, gentamicin decreased GAG storage in the brain and spleen by 32% and 23%, respectively (**Figure 4B, E**). Co-administration of both gentamicin and NMDI-1 decreased excess GAG storage in the brain by 91% compared to untreated *Idua* ^W392X^ controls, resulting in a GAG level that was not statistically different from the level in WT mice. In the spleen, co-treatment with gentamicin and NMDI-1 reduced excess GAGs by 52% compared to untreated controls. Consistent with our previous study [25], NB84 treatment reduced excess GAGs by 40% in both the brain and spleen compared to untreated controls (**Figure 4C, F**). However, no enhancement in GAG reduction was observed when NMDI-1 was co-administered with NB84. Extension of NMDI-1 co-administration with NB84 to six days also did not lead to any further enhancement in GAG reduction (**Figure S6**).

**Figure 5:**
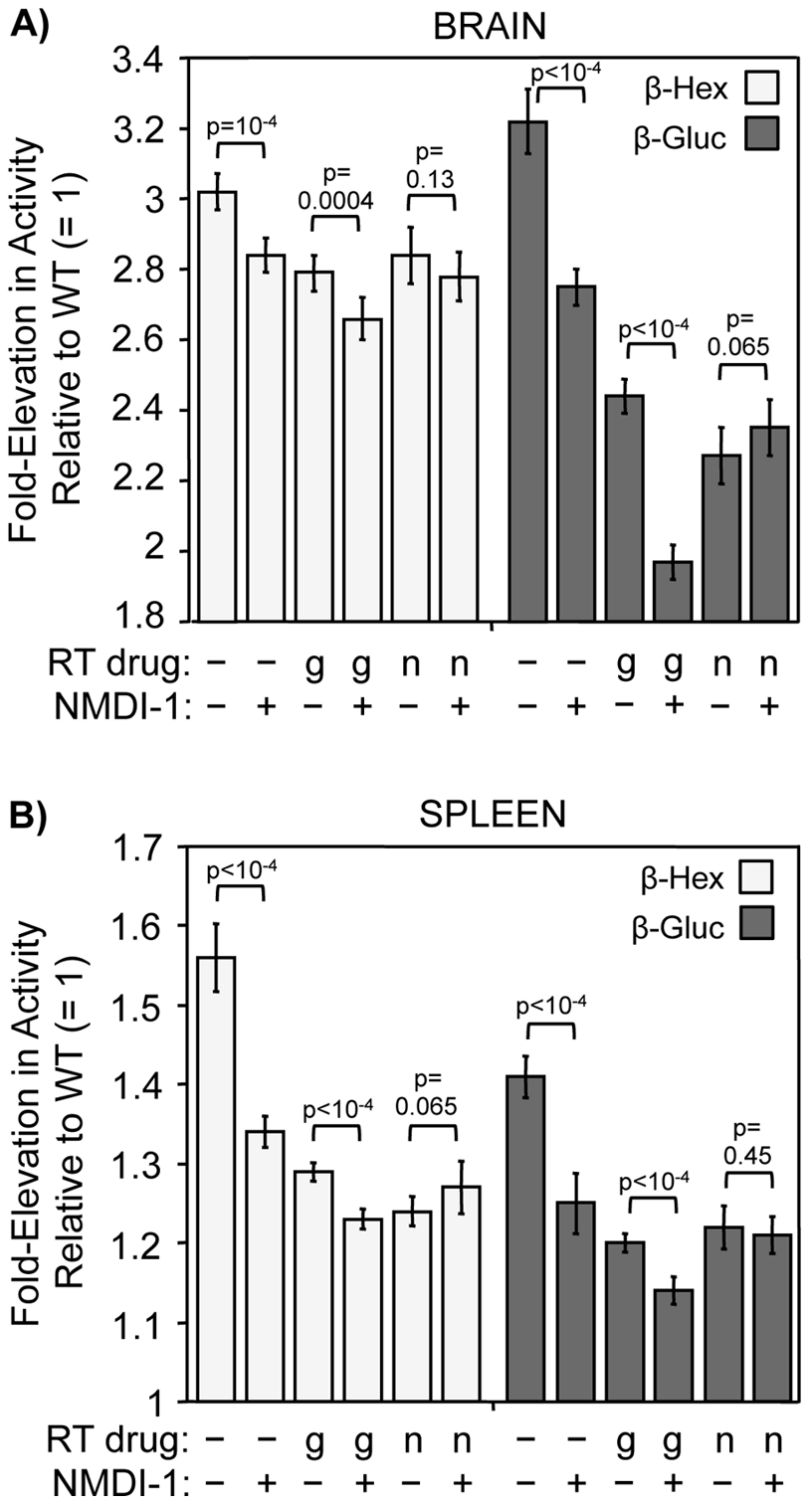
NMDI-1 co-administration with gentamicin alleviates secondary MPS I-H lysosomal markers. *Idua* ^W392X^ mice were administered gentamicin (g) or NB84 (n) for 14 days without (−) or with (+) co-administration of NMDI-1 during the final 3 days of treatment. β-hexosaminidase (β-hex) and β-glucuronidase (β-gluc) enzyme activities were determined in tissue lysates from A) brain and B) spleen. The data are expressed as the fold-elevation in enzyme specific activity measured in treated and untreated *Idua* ^W392X^ mice relative to the level in WT mice, which was normalized to 1. The data are the mean +/− sd of the values obtained from 3–4 mice per treatment group performed with ≥8 replicates (n =  3 or 4). *p* values above the brackets compare mice administered NMDI-1 versus those without NMDI-1 administration within each treatment group.

Previous studies have shown that the activities of several lysosomal enzymes are upregulated in tissues derived from mouse models of several lysosomal storage diseases as a consequence of excess GAG storage [38]. Consistent with this observation, we have shown that β-hexosaminidase and β-glucuronidase activities are increased in homozygous *Idua* ^W392X^ mouse tissues [26]. We next determined whether the reduction in GAG storage in *Idua* ^W392X^ mouse tissues observed with nonsense suppression was sufficient to moderate the upregulation of these lysosomal enzymes. In *Idua* ^W392X^ mice treated with NMDI-1 alone, the activities of both of these enzymes were reduced in the brain and spleen by a small, but significant 5–15% relative to untreated *Idua* ^W392X^ mice (**Figure 5A, B**). Gentamicin treatment reduced both β-hexosaminidase and β-glucuronidase activities in the brain and spleen by 10–15%. NMDI-1 co-treatment with gentamicin resulted in larger 15–35% reductions in the activities of these enzymes. In *Idua* ^W392X^ mice treated with NB84 alone, β-hexosaminidase and β-glucuronidase activities were reduced in the brain and spleen by 10-30% compared to untreated controls. However, co-administration of NMDI-1 with NB84 did not further reduce the activity of either enzyme in the brain or the spleen.

**Figure 6:**
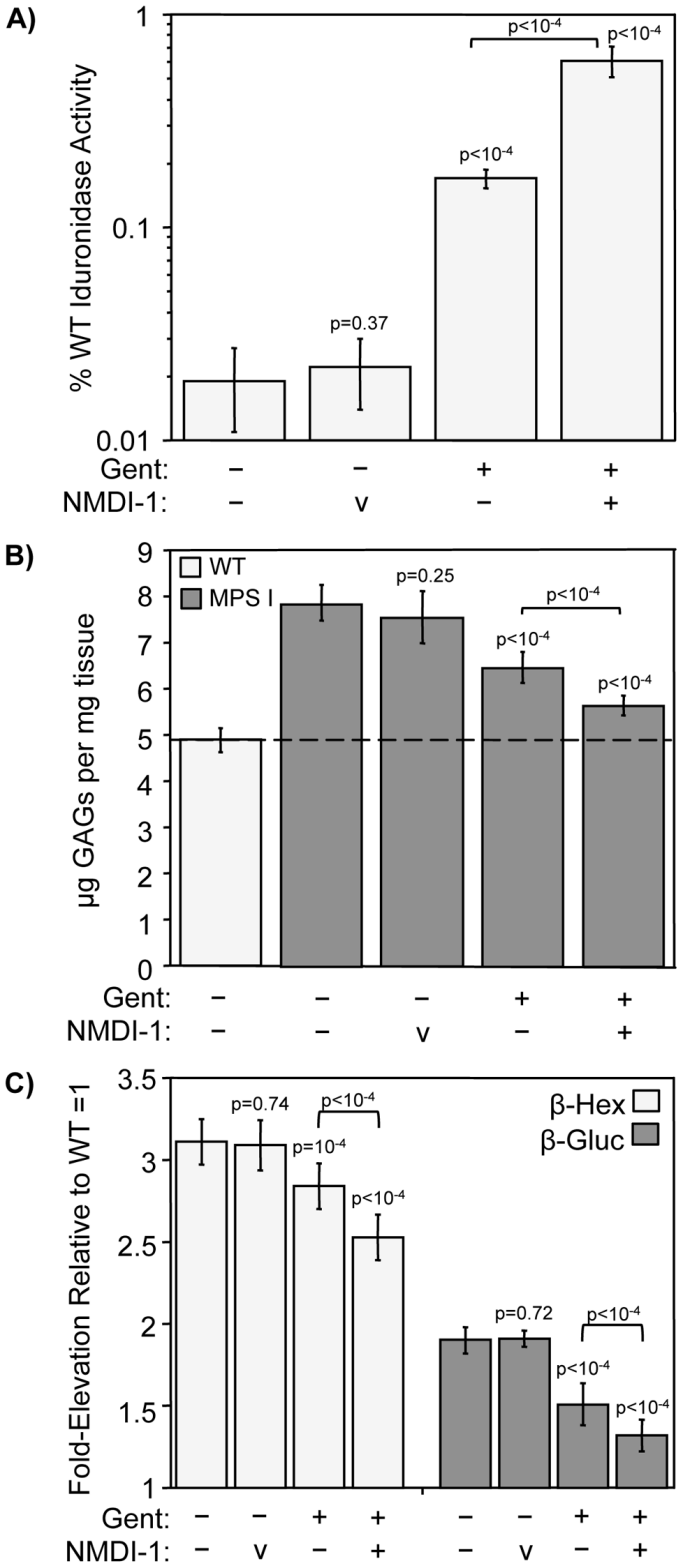
Improvements in MPS I-H biochemical markers are sustained in *Idua* ^W392X^ mice co-administered gentamicin and NMDI-1 for 9-weeks. *Idua* ^W392X^ mice were administered gentamicin 3-times weekly for 9 weeks without (−) or with (+) co-administration of NMDI-1 or vehicle alone (v) two times weekly. MPS I-H biochemical markers were analyzed in brain tissue from treated *Idua* ^W392X^ mice and controls. A) α-L-iduronidase (Idua) specific activity was determined in brain protein lysates. The data are expressed as the specific activity in the mutant mouse tissues relative to WT controls × 100 (% WT Idua Activity). Data are the mean +/− sd of values obtained from 5 mice per group, performed using ≥4 replicates (n = 5). *p* values above the columns compare treated mice to untreated controls, while the *p* values above the brackets compare mice treated with NMDI-1 to those without NMDI-1 treatment. B) Sulfated GAGs were quantitated in defatted, dried brain homogenates from WT mice (light gray bars) and from untreated and treated *Idua* ^W392X^ mice (dark gray bars). GAG levels were quantitated as micrograms GAGs per milligram protein. The dashed line represents the WT GAG level as a reference. Data are expressed as mean +/− sd of 15–18 assays from 5 mice for each experimental group (n = 5). *p* values above the columns compare treated versus untreated W392X mice. *p* values above the brackets compare mice co-treated with both gentamicin and NMDI-1 compared to mice treated with gentamicin alone. C) β-hexosaminidase (β-hex) and β-glucuronidase (β-gluc) enzyme activities were determined in brain protein lysates. The data are expressed as the fold-elevation in enzyme specific activity measured in treated and untreated *Idua* ^W392X^ mice relative to the level in WT mice, which was normalized to 1. The data are the mean +/− sd of the values obtained from 5 mice per treatment group performed with ≥4 replicates (n = 5). *p* values above the brackets compare mice administered NMDI-1 versus those without NMDI-1 administration within each treatment group.

We next investigated whether the improvements observed in MPS I-H biochemical endpoints with short-term gentamicin and NMDI-1 co-administration could be maintained with a longer treatment regimen. To do this, we initiated treatment in mice when they were 3-weeks old and continued treatment until the mice were 12-weeks of age, the same age that mice were evaluated after the 2-week treatment regimen described above. Gentamicin was administered via subcutaneous injections at a dose of 30 mg/kg three times weekly (Monday/Wednesday/Friday). NMDI-1 or vehicle alone was co-administered via subcutaneous injections at a dose of 5 mg/kg twice weekly (Monday/Friday). At the end of the 9-week treatment regimen, we evaluated MPS I-H biochemical endpoints in brain tissues from *Idua* ^W392X^ mice co-treated with gentamicin and NMDI-1 compared to controls. We found that 9-week treatment with gentamicin alone restored 0.17% of normal α-L-iduronidase activity (**Figure 6A**). Co-administration of NMDI-1 with gentamicin resulted in recovery of 0.61% of wild-type α-L-iduronidase activity. The level of restored α-L-iduronidase restored in gentamicin-treated mice reduced GAG storage in the brain by 49% (compared to untreated *Idua* ^W392X^ mice) (**Figure 6B**). GAGs were reduced by 75% in mice treated with both gentamicin and NMDI-1 after the 9-week regimen. This reduction in excess GAG storage corresponded to significant decreases in the activities of the lysosomal enzymes β-hexosaminidase and β-glucuronidase where gentamicin treatment alone reduced the enzymes by 13% and 43%, respectively. Co-administration of NMDI-1 led to decreases of 27% and 64% in β-hexosaminidase and β-glucuronidase, respectively (compared to untreated *Idua* ^W392X^ mice). A direct comparison of the data obtained from the 2-week regimen compared to the 9-week regimen (**Table 1**) reveals that a similar degree of alleviation in MPS I-H biochemical endpoints was observed between the two treatment regimens. This indicates that the benefits of combining NMDI-1 inhibition with suppression therapy can be maintained for extended periods. In addition, no aberrations in mouse behavior, mouse weight, or mouse tissue histology were found with 9-week NMDI-1 treatment.

**Table 1:** Comparison of 2-week and 9-week co-administration of gentamicin with NMDI-1 on MPS I-H biochemical endpoints in *Idua* ^W392X^ mouse brain.

	% WT Iduronidase Activity		% GAG Reduction		% β-HexReduction		% β-Gluc Reduction	
Treatment	2wks	9wks	2wks	9wks	2wks	9wks	2wks	9wks
Untreated	0.018%	0.020%	NA	NA	NA	NA	NA	NA
Gentamicin	0.15%	0.17%	32%	49%	11%	13%	35%	43%
Gent+NMDI1	0.60%	0.61%	91%	75%	18%	27%	56%	64%

## Discussion

From our preliminary screen comparing the efficiency of caffeine and NMDI-1 as NMD attenuators, we found that NMDI-1 attenuates NMD substrate decay more effectively, and at concentrations 1500-fold lower, than caffeine. In addition, NMDI-1 did not inhibit cell growth at incubation times up to 48 hrs at concentrations higher than required to maximally inhibit NMD, while caffeine inhibited cell growth after 8 hrs, even at suboptimal concentrations. The growth inhibition observed with caffeine is likely due to off-target effects on other kinase-mediated signaling pathways. NMDI-1 also did not significantly inhibit protein synthesis or suppress termination at PTCs, suggesting that NMDI-1 attenuates NMD with greater specificity than caffeine. Ellipticine, a compound that has a similar chemical structure to NMDI-1, did not inhibit NMD, but strongly inhibited protein synthesis and cell growth. These results indicate that the structural differences between ellipticine and NMDI-1 are important for NMD inhibition by NMDI-1. These data suggest that NMDI-1 may be a suitable lead compound to establish structure-activity relationships for molecules that mediate NMD inhibition by disrupting interactions between SMG5 and UPF1. Development of effective NMD attenuators may reduce the severity of a broad range of disorders that are exacerbated by NMD [39], [40].

In agreement with our hypothesis, we found that NMD attenuation enhanced PTC suppression efficiency with at least a subset of readthrough drugs. In *Idua* ^W392X^ MEFs treated with both NMDI-1 and suppression drugs, 50% more α-L-iduronidase activity and a 50% greater reduction in GAGs were found compared to cells treated with suppression drugs alone. In *Idua* ^W392X^ mice, treatment with gentamicin, NB84, or NMDI-1 alone significantly increased α-L-iduronidase activity; gentamicin and NB84 restored enough enzymatic activity to significantly decrease excess tissue GAG storage. Importantly, short-term co-administration of NMDI-1 with gentamicin restored more α-L-iduronidase activity than either drug alone. This increase in activity was accompanied by further correction of tissue GAGs and other MPS I-H lysosomal biomarkers. A longer 9-week treatment regimen recapitulated the improvements observed with a short-term treatment regimen, and indicated that the MPS I-H biochemical improvements achieved with co-administration of gentamicin and NMDI-1 could be maintained for extended periods.

Surprisingly, our results indicated that co-administration of NMDI-1 and NB84 to *Idua* ^W392X^ mice did not result in any further improvement in α-L-iduronidase activity, GAG reduction, or additional MPS I-H biomarkers compared to NB84 alone. Extension of NMDI-1 treatment from three to six days concurrent with NB84 treatment also did not provide any further alleviation of these phenotypic endpoints. These results differ from our *in vitro* data showing that NMDI-1 enhanced readthrough mediated by both NB84 and gentamicin by similar amounts in *Idua* ^W392X^ MEFs. Taken together, these results suggest that the lack of synergy between NMDI-1 and NB84 in *Idua* ^W392X^ mice may be attributable to differences in pharmacokinetics, clearance mechanisms, or off-target interactions that occur *in vivo*.

Previous studies have shown that the amount of functional α-L-iduronidase required to improve the MPS I-H phenotype to a milder clinical presentation (designated as Hurler/Scheie or Scheie) may be as low as 0.1–1% of normal [29], [30]. Thus, even the modest levels of enzyme restored in this study (∼0.6% of WT) could lead to a reduction in disease severity. Unlike the well characterized correlation between enzymatic activity and phenotype, the correlation between the level of tissue GAG storage and the severity of the MPS I phenotype has not been as clearly defined. However, one study found that skin fibroblasts from patients with mild forms of MPS I retained 20–70% fewer GAGs than cells from patients with the severe form of MPS I [30]. Based on these values, the reduction in excess tissue GAGs observed in *Idua* ^W392X^ mice treated with gentamicin and NMDI-1 may be sufficient to attenuate the MPS I-H phenotype in at least some tissues. Longer-term treatment will be required to determine the extent that morphological and functional defects of MPS I-H can be alleviated or prevented in the *Idua* ^W392X^ mouse by suppression therapy in combination with NMD attenuation. Since ∼75% of MPS I-H patients carry a nonsense mutation [27], enhancement of suppression therapy with NMD attenuation may represent an effective approach to treat MPS I-H patients. Notably, the combination of gentamicin and NMDI-1 reduced excess brain GAGs in *Idua* ^W392X^ mice to near WT levels. This suggests that both NMDI-1 and gentamicin cross the murine blood brain barrier, and the combination of suppression therapy with NMD attenuation may potentially moderate the neurological defects associated with MPS I-H. This is a particularly important finding, since current MPS I-H treatments such as enzyme replacement therapy do not improve the neurological aspects of the disease [41]. In addition, this approach could potentially be used to treat other neurological diseases attributable to nonsense mutations. These findings support the hypothesis that a synergistic therapeutic enhancement in suppression therapy can be achieved when NMDI-1 is co-administered with some readthrough compounds. Since 11% of all disease-causing gene lesions are nonsense mutations [42], improvements in the efficiency of suppression therapy could provide a therapeutic benefit for many patients who otherwise have only limited treatment options.

Several lines of evidence suggest that NMD perturbation must be approached with caution. Deletion of *UPF1*, *UPF2*, or *SMG1* result in embryonic lethality in mice, suggesting a critical role for NMD and/or NMD factors during mammalian development [43]–[45]. Some NMD factors also have additional cellular functions, including telomere maintenance and regulation of cellular responses to DNA damage and nutrient availability [46]. In addition, NMD factor knock-downs in HeLa cells revealed that 1–10% of the transcriptome is regulated by NMD [34], [47], [48]. However, current evidence also suggests that modest NMD modulation may not lead to adverse effects. First, transgenic mice that constitutively express a dominant-negative UPF1 protein that partially inhibits NMD are viable and develop normally, with the exception of a defect in thymocyte development [49]. Second, NMD factor levels are controlled by an auto-regulatory feedback loop that is dependent upon NMD factor abundance and NMD efficiency, suggesting that NMD is naturally regulated to guard against extreme perturbation [50], [51]. Third, NMD efficiency varies by 2 to 4-fold among individuals within the general population, suggesting that modest fluctuations in NMD efficiency are well tolerated [13], [52], [53]. Taken together, these findings suggest that low-level NMD attenuation imposed after birth may not lead to deleterious effects and may be useful to treat diseases adversely affected by NMD. Importantly, NMDI-1 administration to *Idua* ^W392X^ mice resulted in a ∼1.5-fold increase in endogenous NMD substrates. While no side effects were observed in mice treated with NMDI-1, more extensive longer-term studies are required to determine whether partial NMD inhibition can be maintained and safely tolerated.

## Materials and Methods

### Readthrough drugs and NMD inhibitors

Gentamicin (Hospira, Inc.) was obtained through UAB University Hospital. The Baasov lab (Technion-Israel Institute of Technology) synthesized NB84 [37]. Caffeine (C0750), ellipticine (E3380), cycloheximide (C6255), and G418 (G8168) were purchased from Sigma. NMDI-1 was synthesized using a 15-step synthetic procedure outlined in **Figure S7** that was modified from previous reports [54], [55]. The synthetic modification was carried out on the early part of the synthesis leading to the formation of compound (**7)**, a (*E*) cinnamic acid derivative. The reason for this modification was the unavailability of the starting materials used in the reported procedure. We found that compound **7** can be prepared from 2,5-dimethyl benzaldehyde (**1**) in 6 steps. Conversion of compound **7** to NMDI-1 was carried out according to the published procedure^54,55^. Our synthesis of NMDI-1 started with commercially available 2,5-dimethyl benzaldehyde (**1**). Nitration of compound **1** with a mixture of NaNO_3_ and H_2_SO_4_ provided a mixture of 2,5-dimethyl-3-nitrobenzaldehyde (**2a**) and 3,6-dimethyl-2-nitrobenzaldehyde (**2b**). The mixture of two nitro derivatives was refluxed with malonic acid in the presence of piperidine and pyridine to form the cinnamic acid derivative (**3a**) as the clean product upon crystallization from a mixture CHCl_3_/hexanes. The yield for these two steps is 23%. This reaction also produced the isomeric cinnamic acid (**3b**), which stayed in the mother liquor during crystallization. We did not make an effort to purify and characterize **3b** as it was not needed for our synthesis. Cinnamic acid **3a** was esterified by treatment with MeI in the presence of K_2_CO_3_ in DMF to form the ester **4** in 99% yield. The nitro group present in compound **4** was then reduced to an amino group by using Sn/HCl in MeOH to afford compound **5** in quantitative yield. Acetylation of compound **5** using acetic anhydride in the presence of DMAP and triethyl amine formed the N-acetyl derivative **6** in 74% yield. Alkaline hydrolysis of compound **6** using 3N. NaOH in MeOH afforded the acid **7** in 73% yield. Compound **7** was converted to **NMDI-1** following the reported literature procedure [54], [55] as shown in the **Figure S7**.

### Luciferase Reporter Assays

The *Renilla* luciferase-based NMD reporters were a gift from Dr. Andreas Kulozik (University of Heidelberg, Germany) [32]. The dual luciferase readthrough reporters containing the UAG-W392X (pDB1134) or UGG-W392 (pDB1133) mouse *Idua* codons were previously described [25]. HeLa and HEK293T cells were transfected with the NMD and readthrough reporters, respectively, using Lipofectamine (Invitrogen). HeLa cells were incubated with ellipticine or NMDI-1 (in DMSO) for 20 hrs, with caffeine (in PBS) for 4 hrs, or with cycloheximide (in PBS) for 2 hrs at the indicated concentrations. HEK293Ts were grown in the presence of 1.8 mM gentamicin or 0.72 mM G418 +/− 5uM NMDI-1 for 24 hrs. Luciferase assays were performed with the Dual Luciferase Assay System (Promega) using a Berthold Lumat LB9507 luminometer.

### Protein synthesis assay

HeLa cells cultured in DMEM + 10% FBS growth media until 50% confluent were treated as follows: 7.5 mM caffeine for 4 hrs, 5 μM NMDI-1 for 24 hrs, 5 μM ellipticine for 24 hrs, or 0.81 mM cycloheximide for 2 hrs. Cells were then washed and incubated in DMEM (lacking methionine, cysteine, or glutamine) + 10% dialyzed FBS labeling media for 30 minutes at 37°C. The labeling media was then replaced with a 1∶1 mix of growth and labeling medias containing drugs at the same concentrations as above and 50 μCi/ml ^35^S-methionine (EXPRE 35-S labeling mix, Perkin-Elmer NEG-072) for 2 hr at 37°C. Cells were washed x3 with PBS, scraped in 1.5 mls cold 25% trichloroacetic acid (TCA), and then incubated on ice for 30 minutes. TCA-precipitated protein was filtered through 934-AH Whatman glass fiber filters and ^35^S-methionine protein incorporation was determined using a Wallac 1409 liquid scintillation counter. Unlabeled cells treated under identical conditions were lysed in 750 μl of cold M-Per Protein Reagent (Pierce) to determine total protein concentration. A time course verified that under these experimental conditions, ^35^S-methionine incorporation was linear.

### Reverse transcription quantitative PCR (RT-qPCR)

Total RNA was isolated from mouse tissues and DNase-treated using Ambion RiboPure and Turbo DNA-Free kits, respectively. Polyadenylated RNA was reverse transcribed into cDNA in a 50 μl reaction containing 1 μg of total RNA; 0.5 mg/ml oligo dT; 1.2 mM dNTPs; 40U RNasin (Promega); 10 μl of 5X AMV RT buffer and 40U AMV reverse transcriptase (Fermentas). RT reactions were incubated at 42°C for 1.5 hours, and then heat inactivated at 65°C for 15 minutes. The cDNA was ethanol precipitated and subjected to qPCR in a 25 μl reaction containing 12.5 μl iQ SYBR Green Supermix (Bio-Rad); 0.2 μM of each forward and reverse primer; and cDNA (2 μg for *Atf4*, *Gas5*, *Gapdh*, *Idua*, *Rpl13a* transcripts; 1 μg for 5S rRNA; 0.5 μg for 18S rRNA). The following primers sets (forward  = Pf and reverse  = Pr) were used: *Idua* Pf: 5′-TGACAA TGCCTT CCTGAG CTACCA-3′ and *Idua* Pr: 5′-TGACTG TGAGTA CTGGCT TTCGCA-3′; *Atf4* Pf: 5′-CACAAC ATGACC GAGATGAG-3′ and *Atf4* Pr: 5′-CGAAGT CAAACT CTTTCA GATCC-3′; *Gas5* Pf: 5′-TTTCCG GTCCTT CATTCTGA-3′ and *Gas5* Pr: 5′-TCTTCT ATTTGA GCCTCC ATCCA-3′; *Gapdh* Pf: 5′-TTCCAG TATGAC TCCACT CACGG-3′ and *Gapdh* Pr: 5′-TGAAGA CACCAG TAGACT CCACGAC-3′; 5S rRNA Pf: 5′-GCCATA CCACCC TGAACG-3′ and 5S rRNA Pr: 5′-AGCCTA CAGCAC CCGGTATT-3′; 18S rRNA Pf: 5′-GAAACG GCTACC ACATCCGA-3′ and 18S rRNA Pr: 5′-CACCAG ACTTGC CCTCCA-3′; *Rpl13a* Pf: 5′-ATGACA AGAAAA AGCGGATG-3′ and *Rpl13a* Pr: 5′-CTTTTC TGCCTG TTTCCGTA-3′. qPCR was performed using the CFX96 Real-Time PCR Detection System (Bio-Rad) using a program that included an initial 3 minute denaturation step at 95°C followed by 40 repeated cycles of a 10 second denaturation step at 95°C and a 30 second annealing/extension step at 55°C. Melt curve analysis was initially performed with each primer set to verify that only one gene product was generated from the PCR reactions. A standard curve was performed using each primer set to ensure that under the PCR conditions used, the efficiency ranged between 90–110%. The average quantification cycle (Cq) was determined for each mRNA, and mRNA quantification was performed using the Livak (ΔΔCq) method [56] where 5S rRNA, 18S rRNA, *Gapdh*, and *Rpl13a* served as normalization controls. Cq values among the different samples for the various transcripts ranged from 8–30. qPCR was performed using at least 8–12 replicates for each gene product from each sample.

### Enzymatic assays

Primary mouse embryonic fibroblasts (MEFs) derived from homozygous WT and *Idua* ^W392X^ mice were incubated +/− 1.8 mM gentamicin or 2.9 mM NB84 for 48 hrs +/− 5 μM NMDI-1 for 24 hrs. Assays to determine α-L-iduronidase, β-hexosaminidase, and β-glucuronidase activities and sulfated GAG levels were performed as previously described [25]. Enzyme specific activities were calculated as picomoles of released substrate per milligram of total protein per hour. Enzyme activities remained linear over the incubation times. MEF GAG levels were calculated as nanograms of GAGs per milligram total protein. Tissue GAG levels were calculated as micrograms of GAGs per milligram of defatted, dried tissue using a Blyscan Sulfated Glycosaminoglycan Assay, where chondroitin 6-sulfate was used as a reference. The entire heart, three quarters of the spleen (lacking the anterior extremity), and the entire left brain hemisphere was used to measure tissue GAGs. One quarter of the spleen (the anterior extremity), and half of the right brain cortex, was used to perform enzymatic assays for both control and experimental groups.

### Animal Treatment

Gentamicin and NB84 were dissolved in sterilized PBS and administered to 9–11 week-old mice via once daily subcutaneous injections for 14 days at a 30 mg/kg dose unless otherwise described. NMDI-1 was initially dissolved in DMSO and then diluted 1∶3 with cremophor-EL and administered to mice once daily via subcutaneous injections at a dose of 5 mg/kg for 3 days (during days 12–14 of treatment with gentamicin or NB84) unless otherwise described.

### Ethics Statement

All animal work was conducted according to relevant national and international guidelines. All animal protocols used in this study were reviewed and approved by the UAB IACUC (APN# 120109344).

### Statistics

Statistical analysis was performed using InStat 3.0 software (GraphPad Software, Inc.). All *p* values were calculated using the unpaired two-tail Student's *t*-test.

## Supporting Information

Figure S1 **Action of UPF1 phosphorylation cycle inhibitors.** A) A schematic depicting the UPF1 phosphorylation cycle. The mode of action for caffeine and NMDI-1 on this pathway is shown. B) The structure of NMDI-1. The differences between the NMDI-1 and ellipticine structures are indicated.
(PDF)

Figure S2 **NMDI-1 does not inhibit protein synthesis or suppress PTCs.** A) The effect of various NMD inhibitors on total protein synthesis was determined in HeLa cells. ^35^S-methionine incorporation per microgram of total protein was monitored in untreated cells (−) or cells treated with NMDI-1 (NI1), caffeine (CAF), ellipticine (ELP), or cycloheximide (CHX) as described in the Materials & Methods. The data shown are the average +/− sd of two experiments each performed in triplicate (n = 6). p values above the columns compare treated cells with untreated cells. B) The effect of NMDI-1 on PTC suppression was determined in HEK293T cells expressing luciferase readthrough reporters that are not subject to NMD. The efficiency of PTC suppression +/− the readthrough (RT) drugs gentamicin (GENT) or G418 without NMDI-1 (−) and with NMDI-1 (+) addition is shown and expressed as the normalized firefly activity produced by the PTC reporter relative to WT ×100 (% PTC Readthrough). The data are presented as the mean +/− sd of two experiments each performed in triplicate (n = 6). p values above the brackets compare NMDI-1 treated cells to cells not treated with NMDI-1.
(PDF)

Figure S3 **NMD attenuators increase the abundance of endogenous NMD substrates in MEFs.** RT-qPCR was used to quantify the steady state abundance of endogenous NMD substrates in A) WT and B) *Idua* ^W392X^ MEFs. The data shown are the mean +/− SD (n = 12) of mRNA abundance in treated MEFs relative to untreated MEFs. mRNA abundance was normalized to either 18S rRNA or *Rpl13a*.
(PDF)

Figure S4 **NMDI-1 increases NMD substrate abundance in mouse tissues.** *Idua* ^W392X^ mice were administered 5 mg/kg NMDI-1 for 3 days via once daily subcutaneous injections. After treatment, RNA was isolated from the A) brain, B) heart, and C) spleen and analyzed by RT-qPCR to determine the abundance of *Idua*, *Gas5*, and *Atf4* NMD substrates (normalized to 18S rRNA or *Rpl13a*). The data are expressed as the fold-change in mRNA levels in *Idua* ^W392X^ mice treated with NMDI-1 relative to untreated controls (indicated by the dashed line  = 1). All data are the mean +/− sd of values obtained from 3 mice per group (n = 3).
(PDF)

Figure S5 **NMDI-1 alone does not reduce GAG accumulation in** ***Idua*** **^W392X^ mice.** The micrograms of GAGs per miiligram of defatted, dried tissue were quantitated in A) brain, B) heart, and C) spleen from WT and MPS I-H mice without (−) and with (+) NMDI-1) treatment. The data shown are the mean +/− sd of 6 replicates derived from 3 mice per group (n = 3). p values compare NMDI-1 treated to untreated MPS I-H mice.
(PDF)

Figure S6 **NMDI-1 co-administration with NB84 for 6** **days did not further enhance GAG reduction.** *Idua* ^W392X^ mice were treated with NB84 for 14 days alone, or supplemented with NMDI-1 during the final 3 days or the final 6 days of NB84 administration. The GAG levels were quantitated in A) brain, B) heart, and C) spleen. The data shown for each column is the average +/− sd of the GAG levels in *Idua* ^W392X^ mice relative to WT controls (n = 3). p values compare mice treated with NB84 alone to those co-treated with NMDI-1.
(PDF)

Figure S7 **Scheme for NMDI-1 synthesis.**
(PDF)
