# Supplementary figures and images for: Attenuation of Nonsense-Mediated mRNA Decay Enhances In Vivo Nonsense Suppression
Source: PLoS One. 2013 Apr 10;8(4):e60478. doi: 10.1371/journal.pone.0060478 (PMC3622682; doi:10.1371/journal.pone.0060478)

**A)** UPF1 Phosphorylation Cycle

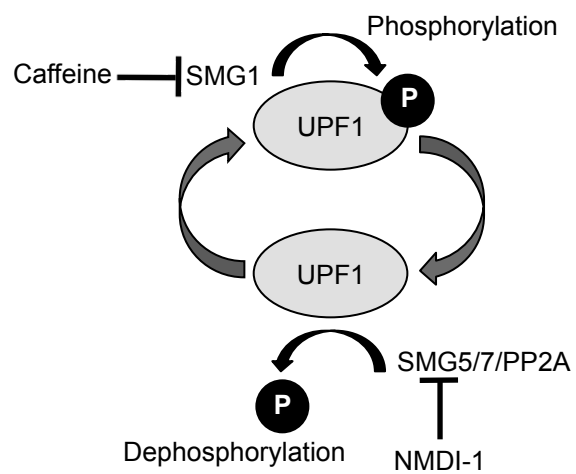

**B)** NMDI-1 Structure

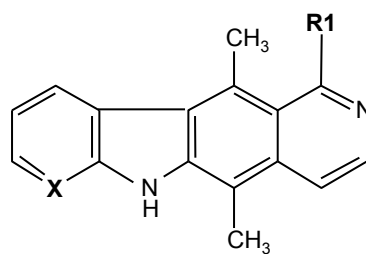

| Drug        | X  | R1 |
|-------------|----|----|
| NMDI-1      | N  | Cl |
| Ellipticine | CH | H  |

Figure S1

Supplement: Figure S1 — Action of UPF1 phosphorylation cycle inhibitors. A) A schematic depicting the UPF1 phosphorylation cycle. The mode of action for caffeine and NMDI-1 on this pathway is shown. B) The structure of NMDI-1. The differences between the NMDI-1 and ellipticine structures are indicated. (PDF) [file pone.0060478.s001.pdf]

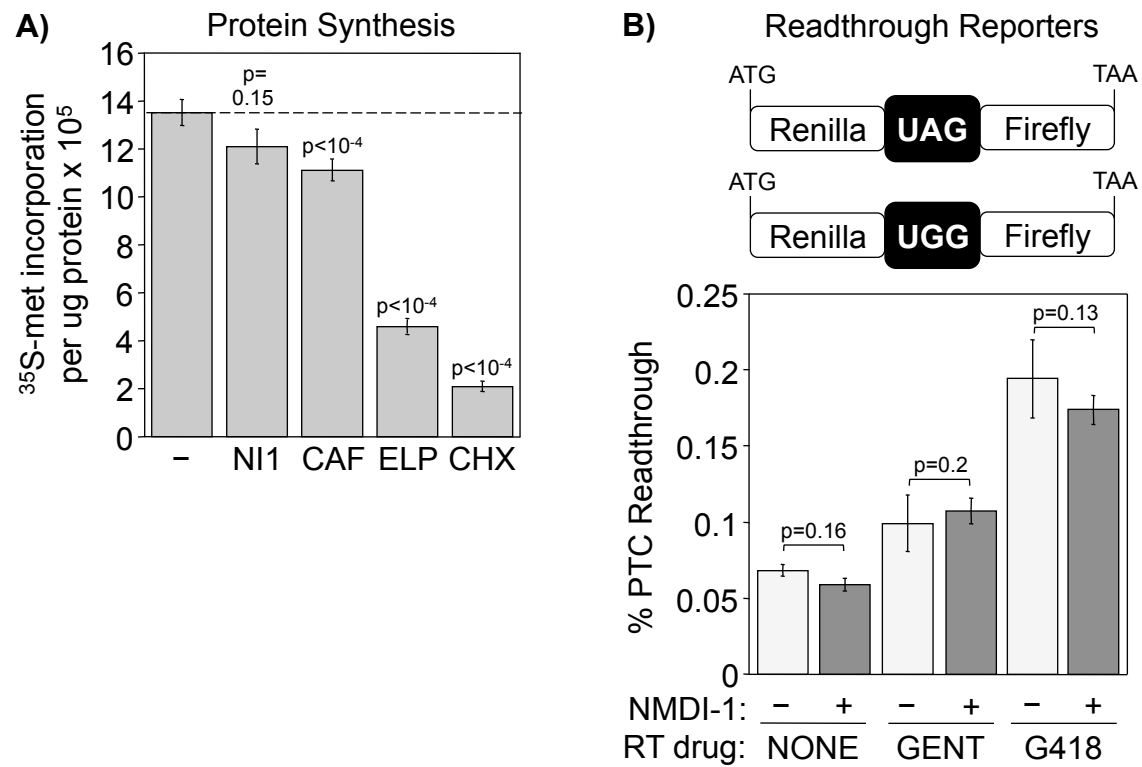

Figure S2

Supplement: Figure S2 — NMDI-1 does not inhibit protein synthesis or suppress PTCs. A) The effect of various NMD inhibitors on total protein synthesis was determined in HeLa cells. 35S-methionine incorporation per microgram of total protein was monitored in untreated cells (−) or cells treated with NMDI-1 (NI1), caffeine (CAF), ellipticine (ELP), or cycloheximide (CHX) as described in the Materials & Methods. The data shown are the average +/− sd of two experiments each performed in triplicate (n = 6). p values above the columns compare treated cells with untreated cells. B) The effect of NMDI-1 on PTC suppression was determined in HEK293T cells expressing luciferase readthrough reporters that are not subject to NMD. The efficiency of PTC suppression +/− the readthrough (RT) drugs gentamicin (GENT) or G418 without NMDI-1 (−) and with NMDI-1 (+) addition is shown and expressed as the normalized firefly activity produced by the PTC reporter relative to WT ×100 (% PTC Readthrough). The data are presented as the mean +/− sd of two experiments each performed in triplicate (n = 6). p values above the brackets compare NMDI-1 treated cells to cells not treated with NMDI-1. (PDF) [file pone.0060478.s002.pdf]

**A) WT MEFs**

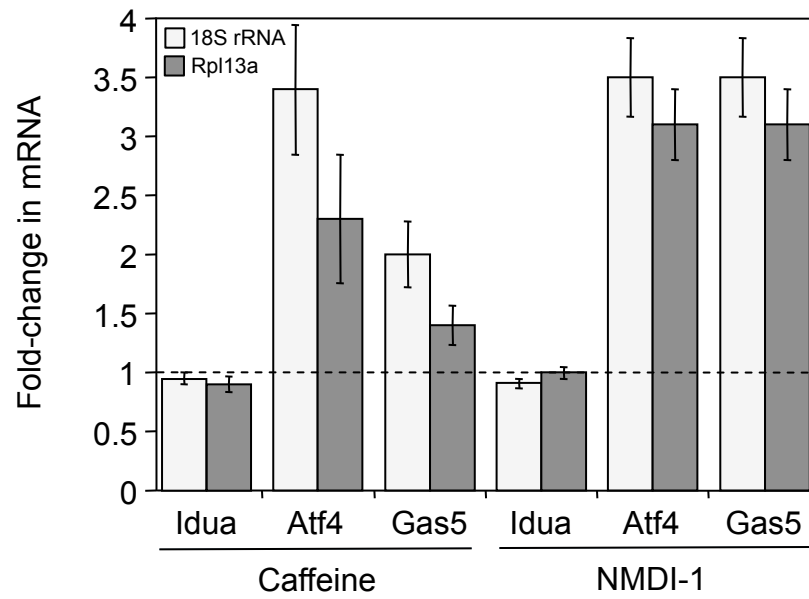

**B) W392X MEFs**

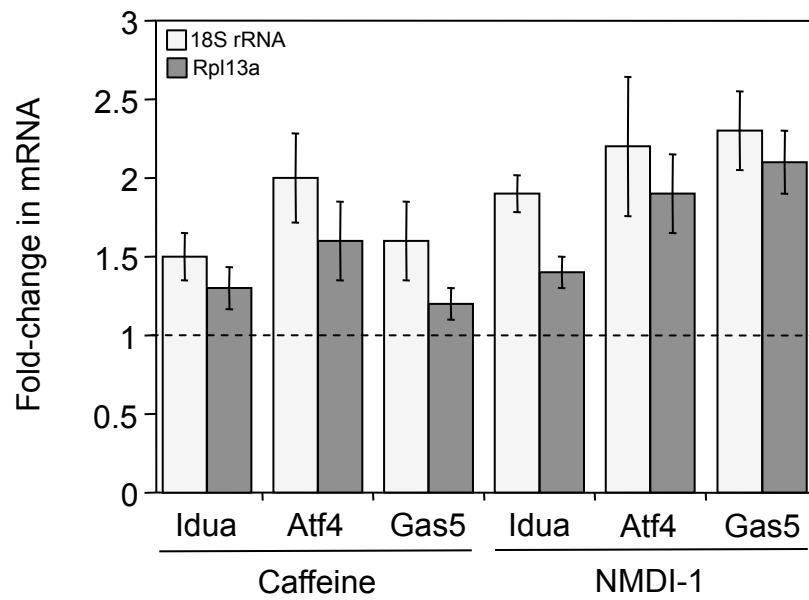

Figure S3

Supplement: Figure S3 — NMD attenuators increase the abundance of endogenous NMD substrates in MEFs. RT-qPCR was used to quantify the steady state abundance of endogenous NMD substrates in A) WT and B) Idua W392X MEFs. The data shown are the mean +/− SD (n = 12) of mRNA abundance in treated MEFs relative to untreated MEFs. mRNA abundance was normalized to either 18S rRNA or Rpl13a. (PDF) [file pone.0060478.s003.pdf]

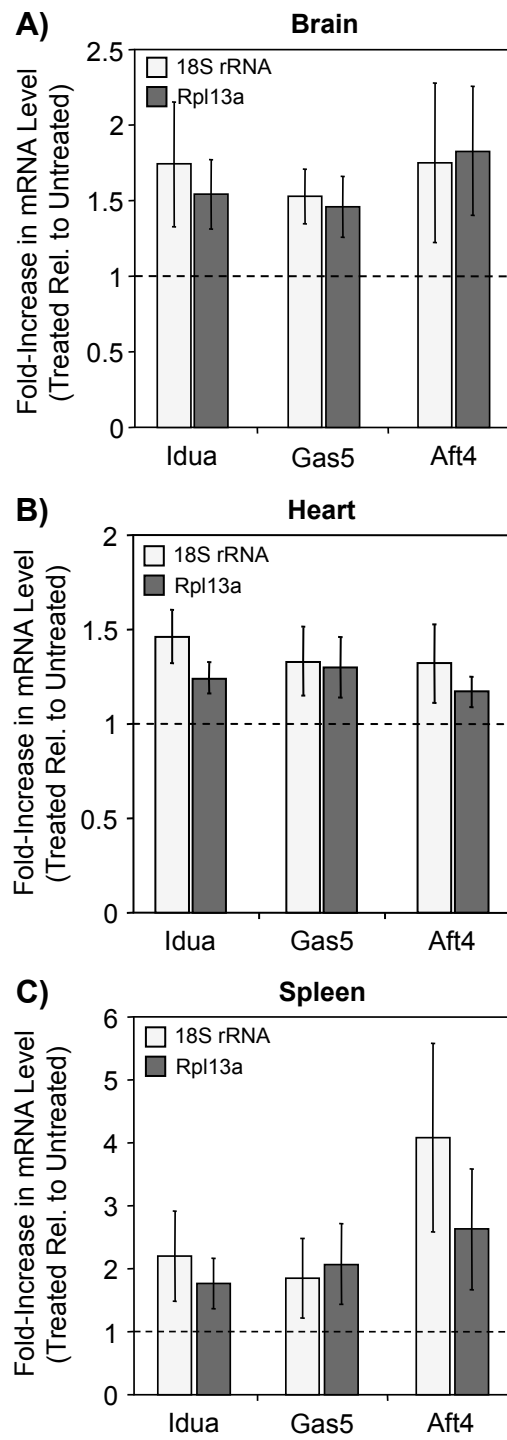

Figure S4

Supplement: Figure S4 — NMDI-1 increases NMD substrate abundance in mouse tissues. Idua W392X mice were administered 5 mg/kg NMDI-1 for 3 days via once daily subcutaneous injections. After treatment, RNA was isolated from the A) brain, B) heart, and C) spleen and analyzed by RT-qPCR to determine the abundance of Idua, Gas5, and Atf4 NMD substrates (normalized to 18S rRNA or Rpl13a). The data are expressed as the fold-change in mRNA levels in Idua W392X mice treated with NMDI-1 relative to untreated controls (indicated by the dashed line = 1). All data are the mean +/− sd of values obtained from 3 mice per group (n = 3). (PDF) [file pone.0060478.s004.pdf]

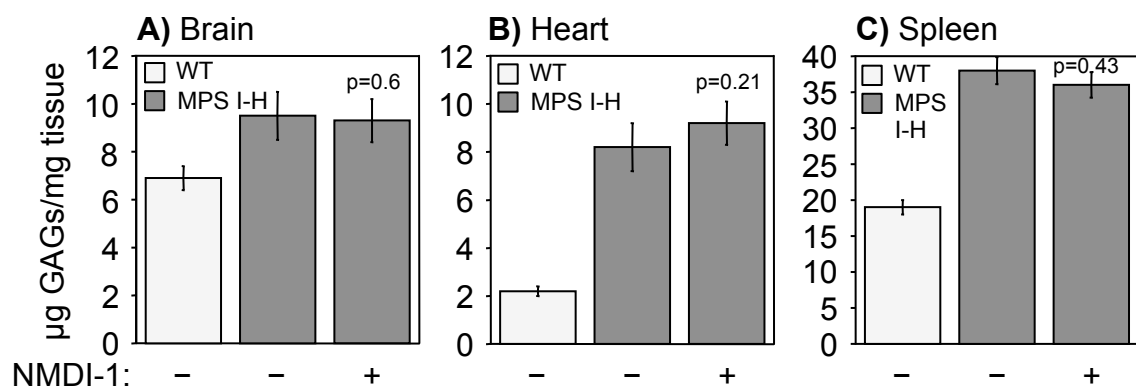

Figure S5

Supplement: Figure S5 — NMDI-1 alone does not reduce GAG accumulation in Idua W392X mice. The micrograms of GAGs per miiligram of defatted, dried tissue were quantitated in A) brain, B) heart, and C) spleen from WT and MPS I-H mice without (−) and with (+) NMDI-1) treatment. The data shown are the mean +/− sd of 6 replicates derived from 3 mice per group (n = 3). p values compare NMDI-1 treated to untreated MPS I-H mice. (PDF) [file pone.0060478.s005.pdf]

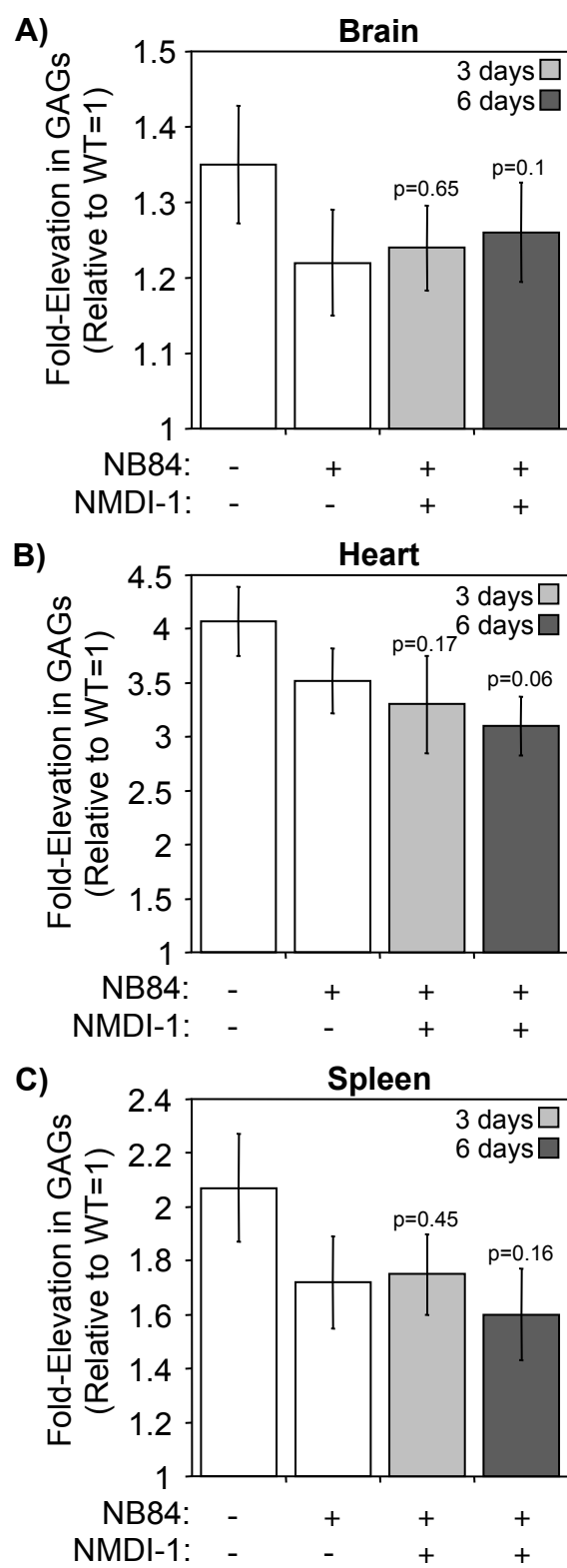

Figure S6

Supplement: Figure S6 — NMDI-1 co-administration with NB84 for 6 days did not further enhance GAG reduction. Idua W392X mice were treated with NB84 for 14 days alone, or supplemented with NMDI-1 during the final 3 days or the final 6 days of NB84 administration. The GAG levels were quantitated in A) brain, B) heart, and C) spleen. The data shown for each column is the average +/− sd of the GAG levels in Idua W392X mice relative to WT controls (n = 3). p values compare mice treated with NB84 alone to those co-treated with NMDI-1. (PDF) [file pone.0060478.s006.pdf]

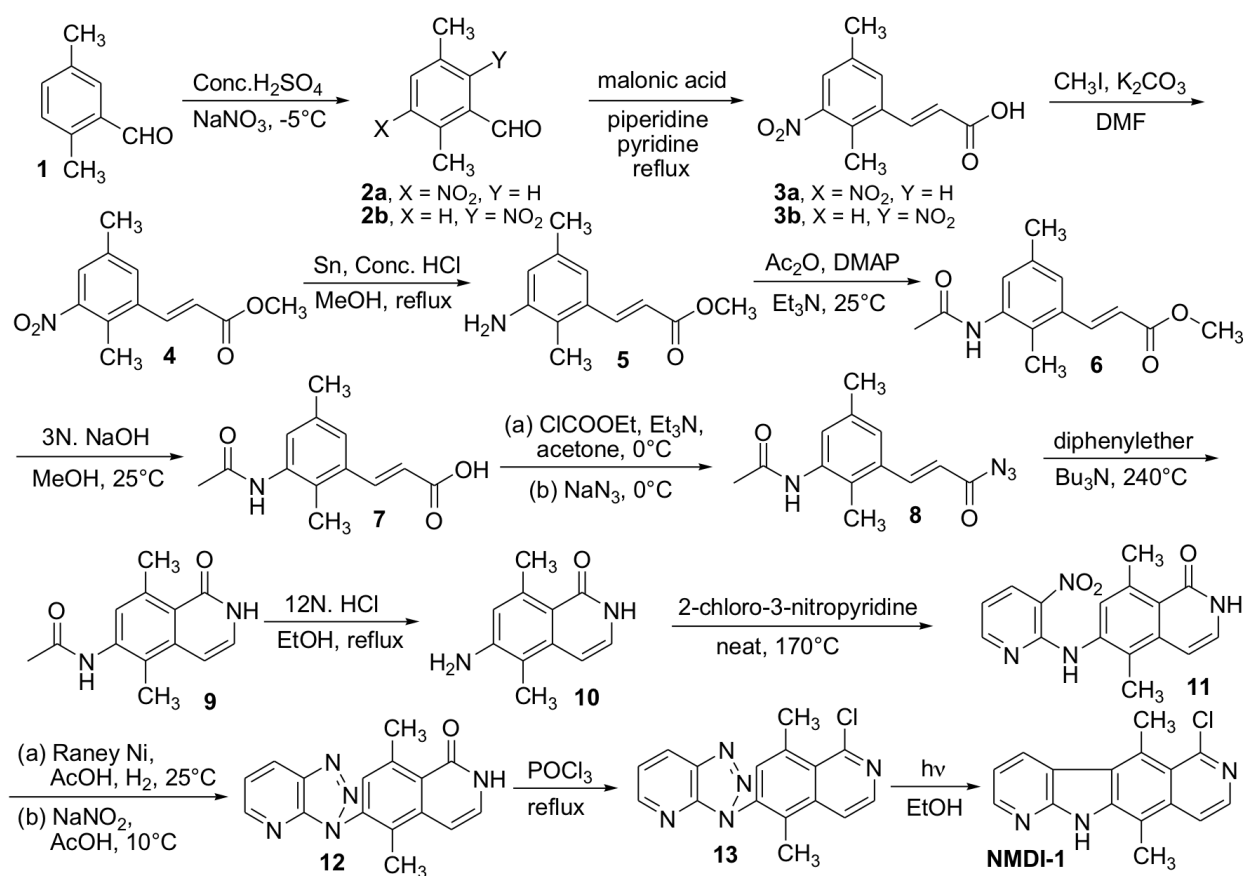

Figure S7

Supplement: Figure S7 — Scheme for NMDI-1 synthesis. (PDF) [file pone.0060478.s007.pdf]
